# Neural activation of regions involved in food reward and cognitive control in young females with anorexia nervosa and atypical anorexia nervosa versus healthy controls

**DOI:** 10.1038/s41398-023-02494-3

**Published:** 2023-06-23

**Authors:** Kamryn T. Eddy, Franziska Plessow, Lauren Breithaupt, Kendra R. Becker, Meghan Slattery, Christopher J. Mancuso, Alyssa M. Izquierdo, Avery L. Van De Water, Danielle L. Kahn, Melissa J. Dreier, Seda Ebrahimi, Thilo Deckersbach, Jennifer J. Thomas, Laura M. Holsen, Madhusmita Misra, Elizabeth A. Lawson

**Affiliations:** 1 Eating Disorders Clinical and Research Program, Massachusetts General Hospital, Boston, MA USA; 2 Department of Psychiatry, Harvard Medical School, Boston, MA USA; 3 Neuroendocrine Unit, Massachusetts General Hospital, Boston, MA USA; 4 Department of Medicine, Harvard Medical School, Boston, MA USA; 5 Division of Women’s Health, Department of Medicine, and Department of Psychiatry, Brigham and Women’s Hospital, Boston, MA USA; 6 Cambridge Eating Disorders Center, Cambridge, MA USA; 7 University of Applied Sciences, Diploma Hochschule, Bad Sooden-Allendorf, Germany; 8 Division of Pediatric Endocrinology, Mass General for Children, Boston, MA USA

**Keywords:** Psychiatric disorders, Neuroscience

## Abstract

Anorexia nervosa (AN) and atypical AN (AtypAN) are complex neurobiological illnesses that typically onset in adolescence with an often treatment-refractory and chronic illness trajectory. Aberrant eating behaviors in this population have been linked to abnormalities in food reward and cognitive control, but prior studies have not examined respective contributions of clinical characteristics and metabolic state. Research is needed to identify specific disruptions and inform novel intervention targets to improve outcomes. Fifty-nine females with AN (*n* = 34) or AtypAN (*n* = 25), ages 10–22 years, all ≤90% expected body weight, and 34 age-matched healthy controls (HC) completed a well-established neuroimaging food cue paradigm fasting and after a standardized meal, and we used ANCOVA models to investigate main and interaction effects of Group and Appetitive State on blood oxygenation level-dependent (BOLD) activation for the contrast of exposure to high-calorie food images minus objects. We found main effects of Group with greater BOLD activation in the dorsal anterior cingulate cortex (dACC), dorsolateral prefrontal cortex (DLPFC), hippocampus, caudate, and putamen for AN/AtypAN versus HC groups, and in the three-group model including AN, AtypAN, and HC (sub-)groups, where differences were primarily driven by greater activation in the AtypAN subgroup versus HC group. We found a main effect of Appetitive State with increased premeal BOLD activation in the hypothalamus, amygdala, nucleus accumbens, and caudate for models that included AN/AtypAN and HC groups, and in BOLD activation in the nucleus accumbens for the model that included AN, AtypAN, and HC (sub-)groups. There were no interaction effects of Group with Appetitive State for any of the models. Our findings demonstrate robust feeding-state independent group effects reflecting greater neural activation of specific regions typically associated with reward and cognitive control processing across AN and AtypAN relative to healthy individuals in this food cue paradigm. Differential activation of specific brain regions in response to the passive viewing of high-calorie food images may underlie restrictive eating behavior in this clinical population.

## Introduction

Anorexia nervosa (AN) and related eating disorders (EDs) are among the most lethal psychiatric conditions with frequent adolescent onset and a protracted illness course [1, 2]. The neurobiology of these complex, multi-system illnesses remains poorly understood. In *DSM-5*, AN and atypical AN (AtypAN) are defined by variable degrees of weight loss and/or low weight achieved through restrictive eating [3]. The shared or divergent pathophysiology underlying degree of weight suppression is almost entirely unknown, particularly during the critical adolescent years when AN and AtypAN typically onset. The study of neural mechanisms that drive aberrant eating behavior during this developmental window of brain maturation is needed to unravel these enigmatic diseases and improve outcomes.

The regulation of eating behavior is complex, involving a metabolic state-dependent balance between brain networks involved in homeostasis, food reward processing, and cognitive control [4–6]. In healthy individuals, these neural networks interact in response to food stimuli. The insula and the hypothalamus receive sensory input that is relayed to the amygdala and orbitofrontal cortex (OFC), which evaluate the subjective reward value of food cues, and then communicate with the striatum, dorsolateral prefrontal cortex (DLPFC), and dorsal anterior cingulate cortex (dACC) to facilitate or suppress reward-related responses [7–10]. In a fasted state, healthy individuals engage homeostatic and reward-related processing in response to food stimuli, reflected in activation in the hypothalamus, anterior insula, amygdala, OFC, hippocampus, and ventral striatum, which drive food intake [4, 11]. By contrast, in a fed state, healthy individuals’ reward circuitry is attenuated, and activation of cognitive control circuits, including DLPFC and dACC, is increased in the presence of food stimuli, which function to modulate appetitive responses and may reduce eating behavior [12–14]. Notably, these regions are activated by visual food cue paradigms [10], and this activation is associated with subsequent eating behavior [15]. It is possible that differential neural activation of the brain regions involved in the processing of food cues in individuals with EDs relative to healthy controls (HC) contributes to the different clinical presentations and illness course in AN and AtypAN.

In individuals with AN, dysregulated eating patterns are associated with an imbalance between reward-related and cognitive control processing regions [5, 16–18], but specific findings vary by study. In response to food cues, activation of neural regions implicated in reward-related processing has been found to be both increased [19] and decreased [20] in this population; while DLPFC and dACC are typically hyperactive in AN, which may reflect recruitment of cognitive control regions [21, 22]. Contributing to the inconsistent findings is the variability in methods across studies, including patient population (e.g., varying illness severity, restricting vs. binge-eating/purging presentation) and metabolic state (e.g., time since last meal) [23]. Furthermore, the neurobiology of individuals with AtypAN has not been carefully studied. No research has examined the respective contribution of both clinical characteristics and metabolic state on neural activation patterns in these AN or AtypAN patient populations.

We investigated neural activation of brain regions known to be involved in appetite and food intake using functional magnetic resonance imaging (fMRI) to evaluate blood oxygenation level-dependent (BOLD) activation to the passive viewing of images of high-calorie foods (vs. objects) fasting (premeal) and postmeal in adolescent and young adult females with AN and AtypAN. While recent systematic reviews [24, 25] suggest similarities between AN and AtypAN, whether differences in neurobiology underlie phenotypic differences in weight presentation has not been studied. Thus, we elected to focus on the combined ED sample versus HC, and then perform a secondary analysis splitting the ED group into AN and AtypAN subgroups in order to better understand where between-group differences lie and whether they are driven by low weight. First, for the two-group comparison, we predicted main group effects with increased BOLD activation in the DLPFC and dACC in the ED versus HC groups. As previous studies have shown both increases and decreases in reward-related BOLD activation in individuals with low-weight EDs relative to HC [19, 20], we hypothesized non-directional main group differences in BOLD activation in the anterior insula, hypothalamus, amygdala, OFC, hippocampus, and ventral striatum. For the three-group comparison, we hypothesized main effects of Group with (1) greater activation of the anterior insula, hypothalamus, amygdala, OFC, hippocampus, and ventral striatum in individuals with AtypAN relative to the AN subgroup and reduced activation in the AN subgroup relative to the HC group, and (2) increased activation of DLPFC and dACC in the AN and AtypAN subgroups relative to the HC group. Second, we hypothesized a main effect of Appetitive State across all (sub-)groups with attenuation of neural activation across these cognitive control and reward-related neural regions from premeal to postmeal. Third, we hypothesized an interaction between Group and Appetitive State such that there would be less attenuation in the reward-related brain regions in those with AtypAN relative to the AN subgroup. These findings could suggest neural bases for pathological restrictive eating and help to explain differences in presentation, namely degree of low weight, if relatively higher weight is related to greater sustained postmeal food reward-related responsiveness, and ultimately guide the development of individualized treatment strategies.

## Materials and methods

### Participants

Fifty-nine adolescent and young adult females (ages 10–22 years) with low-weight EDs (≤90% of expected body weight determined by the 50th percentile body mass index [BMI] for age, bone age, or height percentile by Centers for Disease Control and Prevention [CDC] charts) and 34 age-matched HC were included in this observational study. ED participants were diagnosed with *DSM-5* AN (*n* = 34) or AtypAN (*n* = 25), differentiated by weight: AN had a BMI percentile of ≤10 (for those <18 years old) or a BMI ≤ 18.5 kg/m^2^ (for those ≥18 years); *DSM-5* AtypAN was defined as meeting all criteria for AN but without the commensurate degree of low weight. The resulting BMI percentile range in our sample of females <18 years was <1–10 for the AN group, 11–37 for the AtypAN group, and 20–84 for HC; the observed BMI range for participants ≥18 years in our study was 14.65–18.37 kg/m^2^ for the AN group, 18.59–20.90 kg/m^2^ for the AtypAN group, and 19.40–24.40 kg/m^2^ for the HC group. They were further characterized as restricting-type (*n* = 43) or BP-type (≥ 3 binge-eating and/or purging behaviors per month over the previous 3 months; *n* = 16) split up as follows: 25 participants with AN and restricting-type, 9 participants with AN and BP-type, 18 participants with AtypAN and restricting-type, and 7 participants with AtypAN and BP-type. (An additional exploratory analysis contrasting HC, restrictive-type AN, and BP-type AN (sub-)groups is included in the Supplementary Materials.) Diagnoses were conferred via the Kiddie Schedule for Affective Disorders and Schizophrenia-Present and Lifetime (K-SADS-PL; [26]) and confirmed via symptom counts from the Eating Disorder Examination (EDE) version 17.0 [27]. HC females were included if they were between the 25th–85th BMI percentiles for age, reported regular menses (if ≥2 years postmenarcheal), had no pubertal delay (i.e., menarche at >16 years or thelarche at >13 years), engaged in <10 h of exercise or <25 miles of running per week in the preceding three months, and had no lifetime history of any psychiatric disorder as determined by the K-SADS-PL. Exclusion criteria for all participants included the use of systemic hormones, pregnancy, breastfeeding within eight weeks of the baseline visit, a history of psychosis, active substance abuse, hematocrit <30%, potassium level <3 mmol/L, and a history of gastrointestinal tract surgery or other conditions leading to low weight and/or endocrine alterations.

This research was approved by the Institutional Review Board of Mass General Brigham and performed in accordance with the Declaration of Helsinki. Written informed consent was obtained from participants ≥18 years and parents of participants <18 years old, and assent was obtained from participants <18 years old. Visits took place at Massachusetts General Hospital and the Athinoula A. Martinos Center for Biomedical Imaging.

### Overview of procedures

Participants arrived following a 10-h overnight fast and completed an fMRI session, both before and after an ~400-kcal breakfast meal standardized for macronutrient content (~20% protein, 20% fat, 60% carbohydrates) and consumed over 15 min [23]; and an EDE interview. A portion of the clinical characteristics has been previously published [28–31]. However, no BOLD activation data have been reported.

### Functional MRI paradigm

Participants completed two fMRI scanning sessions (premeal; postmeal) using a well-established food cue paradigm involving passive viewing of food and non-food pictures [20, 32]. In a block design, participants viewed 100 high-calorie food stimuli (50 savory, 50 sweet), 100 low-calorie food stimuli, 100 objects, and 100 fixation stimuli, each presented for 3 s using Presentation software (Neurobehavioral Systems, Albany, CA). In a fasted state, healthy individuals rate the high-calorie foods as more appetizing/pleasant than the objects; in a fed state, appetitive ratings of the categories do not differ [20], providing proof-of-concept that the used high-calorie food images are palatable. Stimuli were projected onto a screen positioned at the rear of the magnet and viewed via a coil-mounted mirror. Participants were instructed to press a button when pictures changed to ensure attention to the task. A total of five 4-min runs with five images in each block and 16 blocks in each run were completed. At each session, unique stimuli (new images) were presented to minimize the risk of habituation to the stimuli, and the order of each image category within each run was pseudorandomized. This food cue task was chosen because (1) it reliably activates the anterior insula, hypothalamus, amygdala, OFC, hippocampus, ventral striatum, and DLPFC [10, 20, 32], which are known to be involved in eating behavior [15]; (2) in HC, neural activation in the anterior insula, amygdala, OFC, and striatum is associated with levels of orexigenic ghrelin, the primary peripheral hormone associated with food-seeking and appetite [33–35] and with subsequent food intake [12–14]; and (3) across groups, neural activation in these regions attenuates from premeal to postmeal, suggesting its responsiveness to fasted/fed state [20].

### Functional MRI data acquisition

Whole-brain fMRI data were acquired using a Siemens 3T Trio scanner (Siemens, Erlangen, Germany) equipped with a 12-channel head coil. Head movements were restricted with foam cushions. Functional and structural sequence details are provided in the Supplementary Materials.

### Data processing and statistical analysis

Functional MRI data were preprocessed and analyzed using Statistical Parametric Mapping v12 (SPM12; Wellcome Department of Cognitive Neurology, London, UK; www.fil.ion.ucl.ac.uk/spm). Standard preprocessing procedures and subject-level modeling are detailed in the Supplementary Materials.

Following subject-level analysis, the high-calorie food versus object contrast was introduced into group-level designs according to our hypotheses, and group-level contrast maps were generated for each model to test main effects. Notably, because we were interested in what drives maladaptive undereating in those with EDs, we elected to focus on the contrast of neural activation in response to high-calorie food images versus objects, and our study hypotheses were based on this contrast. This rationale built on prior evidence of robust neural activation in the contrast of high-calorie food images to non-food images in healthy controls [10] and positive relationships between ghrelin and neural activation to high-calorie food versus objects [35]. We contrasted neural activation in response to high-calorie food images with neural responses to objects (rather than a fixation stimulus) to control for all other aspects of visual processing of complex pictures. Please note that food and non-food images were matched for color, brightness, saliency, etc. We first examined the overall response to high-calorie food cues across subjects, including the within-subjects factor Appetitive State (premeal, postmeal), with subjects from both groups (HC, ED) included in each model. As age and estrogen status can impact these activations, age and estradiol levels (log_10_-transformed to approximate normal distribution) on the day of testing were included as covariates in all analyses. To test our hypotheses, we used repeated-measure analyses of covariance (ANCOVAs) with the between-subjects factor Group (Model 1: HC vs. ED; Model 2: HC vs. AN vs. AtypAN; Model 3: HC vs. Restricting-type vs. BP-type [Supplementary Materials]) and within-subjects factor Appetitive State (premeal, postmeal) in response to high-calorie food cues. Post hoc tests (Bonferroni-corrected) for directionality of the observed main effects of Group for Models 2 and 3 were performed using SPSS Statistics (version 28; IBM, Armonk, NY, USA) based on estimated marginal means. Significance was defined as *P* < 0.05.

In SPM12, for each model, main effects and interactions were examined using the small-volume correction approach, restricting voxel-wise analyses to voxels within a priori regions of interest (ROIs). Predefined ROIs were anterior insula, hypothalamus, amygdala, OFC, hippocampus, nucleus accumbens (NAcc), caudate, and putamen as reward-related ROIs and dACC and DLPFC as cognitive control-related ROIs. The anatomical ROIs were defined using the Automated Anatomical Labeling atlas version 3 (AAL3; [36]). Within each ROI, we report clusters that (a) were initially significant at *P* < 0.05 uncorrected, (b) met or exceeded an extent threshold of *k* = 5 for the NAcc and *k* = 20 for all other ROIs, and (c) additionally met the peak-level threshold of *P* < 0.05, FWE-corrected for the ROI. For clusters in a priori ROIs reaching statistical significance for the above models, parameter estimates were extracted with the REX toolbox [37] for visual display and plotting (see Supplementary Materials). In addition to hypotheses for a priori ROIs, main effects and interactions in whole-brain activation to high-calorie foods (i.e., not restricted to a priori ROI masks) were examined at a conservative threshold to guard against spurious findings: significant at *P* < 0.001, uncorrected and met a whole-brain cluster-level threshold of *P* < 0.05, FWE-corrected (Supplementary Materials). Finally, although we did not have hypotheses regarding responsivity to low-calorie foods, for each of the three models described above, we used ANCOVAs to examine main effects of and interactions between Group and Appetitive State for the low-calorie foods versus objects contrast at the whole-brain level using the same whole-brain threshold (*P* < 0.001, uncorrected; whole-brain cluster-level threshold of *P* < 0.05, FWE-corrected; methods and results are reported in the Supplementary Materials).

## Results

### Participant characteristics

Participant demographic and clinical characteristics are presented in Table 1. As expected, individuals with AN and AtypAN presented with lower weight and higher ED psychopathology than HC. By definition, individuals with AN had lower weight than AtypAN. The severity of ED psychopathology did not differ between AN and AtypAN subgroups. Compared to HC, the ED group had lower estradiol levels on the day of testing, which was driven by the AN subgroup (Table 1).

**Table 1:** Participant characteristics.

Characteristic	HC group (*n* = 34)		ED group (*n* = 59)		AN subgroup (*n* = 34)		AtypAN subgroup (*n* = 25)		HC versus ED groups		HC, AN, and AtypAN (sub-)groups	
	*n*	Mean ± SD/*n* (%)	*n*	Mean ± SD/*n* (%)	*n*	Mean ± SD/*n* (%)	*n*	Mean ± SD/*n* (%)	*P*	Cohen’s *d*	*P*	η_*P*_^2^
Age (years)	34	18.2 ± 2.9	59	19.1 ± 2.6	34	19.5 ± 2.2	25	18.5 ± 3.1	0.125	0.33	0.132	0.04
Race	34		59		34		25					
American Indian/Alaska Native		0 (0.0)		0 (0.0)		0 (0.0)		0 (0.0)				
Black/African American		0 (0.0)		0 (0.0)		0 (0.0)		0 (0.0)				
Asian		5 (14.7)		10 (16.9)		7 (20.6)		3 (12.0)				
White		28 (82.4)		48 (81.4)		26 (76.5)		22 (88.0)				
Other		1 (2.9)		1 (1.7)		1 (2.9)		0 (0.0)	1.000		0.867	
Ethnicity	34		59		34		25					
Hispanic/Latino		1 (2.9)		4 (6.8)		4 (11.8)		0 (0.0)				
Non-Hispanic/Latino		33 (97.1)		55 (93.2)		30 (88.2)		25 (100.0)	0.649		0.183	
Time since self-diagnosis (years)	n/a	n/a	58	4.4 ± 3.6	33	4.9 ± 3.5	25	3.8 ± 3.6	n/a	n/a	n/a	n/a
BMI (kg/m^2^)	34	21.6 ± 1.7	59	17.6 ± 1.4	34	16.8 ± 1.1	25	18.8 ± 1.1	**<0.001**	2.98	**<0.001**^a,b,c^	0.72
BMI z-score (if <20 years of age)	24	0.2 ± 0.5	32	−1.5 ± 0.8	17	−2.1 ± 0.6	15	−0.8 ± 0.3	**<0.001**	2.41	**<0.001**^a,b,c^	0.79
EDE												
Restraint	34	0.0 ± 0.0	59	2.9 ± 1.5	34	2.7 ± 1.6	25	3.0 ± 1.5	**<0.001**	2.35	**<0.001**^a,b^	0.57
Eating Concern	34	0.0 ± 0.0	58	1.9 ± 1.4	34	1.7 ± 1.4	24	2.2 ± 1.4	**<0.001**	1.75	**<0.001**^a,b^	0.44
Shape Concern	34	0.1 ± 0.2	59	3.3 ± 2.0	34	3.1 ± 2.0	25	3.6 ± 1.9	**<0.001**	2.05	**<0.001**^a,b^	0.51
Weight Concern	34	0.04 ± 0.1	59	3.1 ± 1.8	34	2.9 ± 1.8	25	3.3 ± 1.9	**<0.001**	2.06	**<0.001**^a,b^	0.51
Global Score	34	0.03 ± 0.1	58	2.8 ± 1.5	34	2.6 ± 1.5	24	3.0 ± 1.5	**<0.001**	2.32	**<0.001**^a,b^	0.57
Estradiol (pg/mL)	34	61.3 ± 50.9	59	41.1 ± 46.5	34	36.8 ± 39.5	25	47.1 ± 55.0				
Log_10_-estradiol	34	1.7 ± 0.3	59	1.3 ± 0.6	34	1.3 ± 0.7	25	1.4 ± 0.6	**<0.001**	0.65	**0.010**^a^	0.10

*AN* anorexia nervosa, *AtypAN* atypical AN, *BMI* body mass index, *EDE* Eating Disorder Examination, *HC* healthy control.

*Note*. Significant *P* values are in bold. ^a^HC group≠AN subgroup, *P* < 0.05; ^b^HC group≠AtypAN subgroup, *P* < 0.05; ^c^AN subgroup≠AtypAN subgroup, *P* < 0.05.

### Model 1: ED group compared to HC

**Fig. 1:**
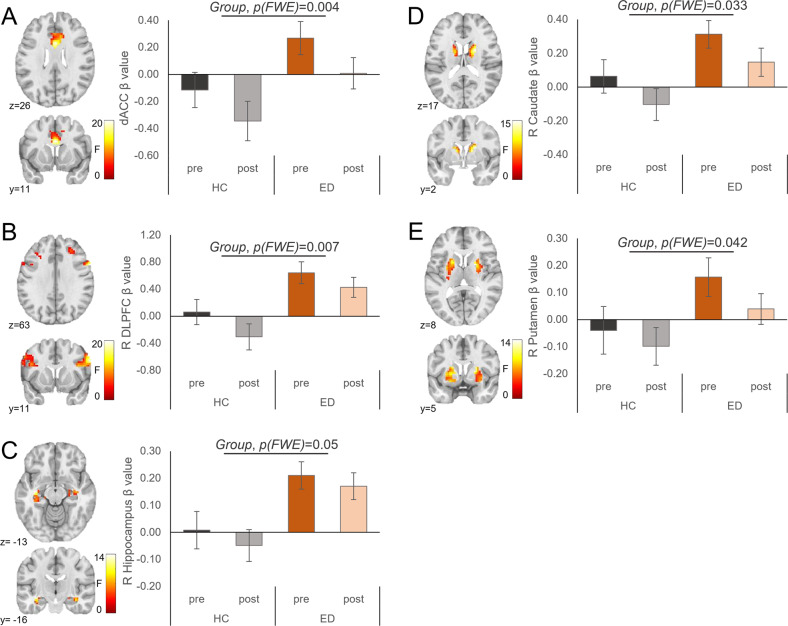
Results of Model 1 showing significant main effects of Group (healthy control [HC]; eating disorder [ED]) in blood oxygenation level-dependent (BOLD) activation to high-calorie foods versus objects in a priori regions of interest (ROIs). BOLD activation differed between groups (HC: *n* = 34; ED: *n* = 59) in the (**A**) dorsal anterior cingulate cortex (dACC), (**B**) right dorsolateral prefrontal cortex (DLPFC), (**C**) left hippocampus, (**D**) right caudate, and (**E**) right putamen. The *F* scale and *P* values reflect the main effect of Group from the 2 (Group) × 2 (Appetitive State) analysis of covariance (with age and estradiol levels at the time of testing as covariates). Statistical thresholding reflects small-volume correction (SVC) within an anatomically-defined bilateral ROI at *P*(FWE-corrected) < 0.05. Statistical maps for BOLD activation are overlaid on a normalized canonical image (Montreal Neurological Institute [MNI ICBM 152 nonlinear asymmetric T1 template) with SPM color map corresponding to the relative *F* value. Coordinates (*y*, *z*) are presented in MNI space, with *y* corresponding to the coronal plane and *z* to the axial plane. Bar graph (right) depicts mean *β* values within each cluster for each group and Appetitive State ± SEM.

**Fig. 2:**
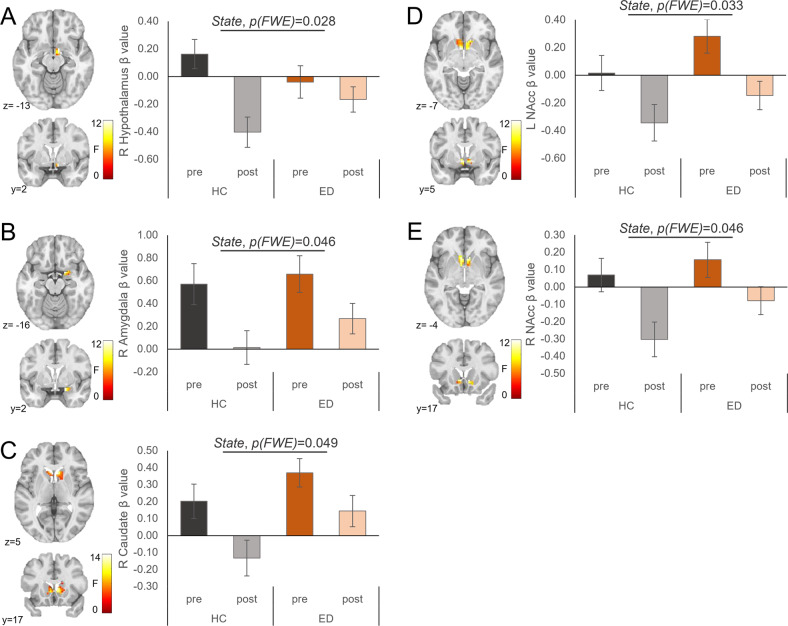
Results of Model 1 showing significant main effects of Appetitive State (premeal [pre]; postmeal [post]) in blood oxygenation level-dependent (BOLD) activation to high-calorie foods versus objects in a priori regions of interest (ROIs). BOLD activation differed between Appetitive States in the (**A**) right hypothalamus, (**B**) right amygdala, (**C**) right caudate, and (**D**) left and (**E**) right nucleus accumbens (NAcc). The *F* scale and *P* values reflect the main effect of the Appetitive State from the 2 (Group) × 2 (Appetitive State) analysis of covariance (with age and estradiol levels at the time of testing as covariates). Statistical thresholding reflects small-volume correction (SVC) within an anatomically-defined bilateral ROI at *P*(FWE-corrected) < 0.05. Statistical maps for BOLD activation are overlaid on a normalized canonical image (Montreal Neurological Institute [MNI] ICBM 152 nonlinear asymmetric T1 template) with SPM color map corresponding to relative *F* value. Coordinates (*y*, *z*) are presented in MNI space, with *y* corresponding to the coronal plane and *z* to the axial plane. Bar graph (right) depicts mean *β* values within each cluster for each (sub-)group and Appetitive State ± SEM.

We found the main effects of Group for BOLD activation in the dACC, right DLPFC, left hippocampus, right caudate, and right putamen (no significant effects in the anterior insula, hypothalamus, amygdala, OFC, or NAcc); and main effects of Appetitive State for BOLD activation in the right hypothalamus, right amygdala, bilateral nucleus accumbens, and right caudate (no significant effects in the anterior insula, OFC, hippocampus, putamen, dACC, or DLPFC). We found no Group by Appetitive State interaction effects in any a priori ROIs (Table 2). Figure 1 depicts between-group differences in BOLD activation for those regions that differed by the group; across regions, BOLD activation was greater in the ED group relative to the HC group. Figure 2 depicts differences in BOLD activation between appetitive states; across regions, BOLD activation attenuated post meal.

**Table 2:** Significant main effects and interactions of Group and Appetitive State (premeal, postmeal) for the two primary models (1: Healthy control [HC] vs. eating disorder [ED] groups; 2: HC vs. anorexia nervosa [AN] vs. atypical AN [AtypAN] [sub-]groups) for blood oxygenation level-dependent (BOLD) activation to high-calorie foods versus objects in a priori primary and secondary regions of interest with age and estradiol levels at the time of testing as covariates.

	R/L^a^	Peak *F* value	*k*(E)^b^	*P* (FWE_corr_)^c^	*x*^d^	*y*	*z*	Also meets whole-brain thresholds
*Model 1: HC (n* *=* *34) versus ED (n* *=* *59) groups*								
Main effect of Group								
dACC	–	19.99	403	0.004	0	11	26	x
DLPFC	R	21.33	109	0.007	63	11	32	x
Hippocampus	L	14.49	88	0.05	−36	−16	−13	
Caudate	R	15.1	164	0.033	21	2	17	
Putamen	R	14.84	187	0.042	21	5	8	x
Main effect of Appetitive State								
Hypothalamus	R	11.36	13	0.028	9	2	−13	
Amygdala	R	11.82	16	0.046	18	2	−16	
NAcc	L	11.83	36	0.033	−6	5	−7	
	R	11	37	0.046	9	17	−4	
Caudate	R	14.19	78	0.049	6	17	5	
Group × Appetitive State interaction								
No significant clusters								
*Model 2: HC (n* *=* *34), AN (n* *=* *34), and AtypAN (n* *=* *25) (sub-)groups*								
Main effect of Group								
dACC	–	10.44	317	0.014	0	11	26	x
DLPFC	R	11.41	118	0.021	63	11	32	
Hippocampus	R	9.6	150	0.035	36	−19	−19	
Caudate	R	9.51	156	0.031	21	2	17	
Putamen	R	9.88	265	0.027	27	−7	11	
Main effect of Appetitive State								
NAcc	L	12.56	35	0.024	−6	5	−7	
Group × Appetitive State interaction								
No significant clusters								

*dACC* dorsal anterior cingulate cortex, *DLPFC* dorsolateral prefrontal cortex, *NAcc* nucleus accumbens.

*Note*. ^a^R/L denotes hemisphere in which peak voxel within each cluster was localized. ^b^Cluster size (contiguous voxels). ^c^Statistical significance was assessed at *P* < 0.05 FWE-corrected using small-volume correction with a minimum cluster size of *k* = 5 in the nucleus accumbens and *k* = 20 in all other regions of interest. ^d^Coordinates are presented in Montreal Neurological Institute (MNI) space.

### Model 2: HC, AN, and atypical AN

**Fig. 3:**
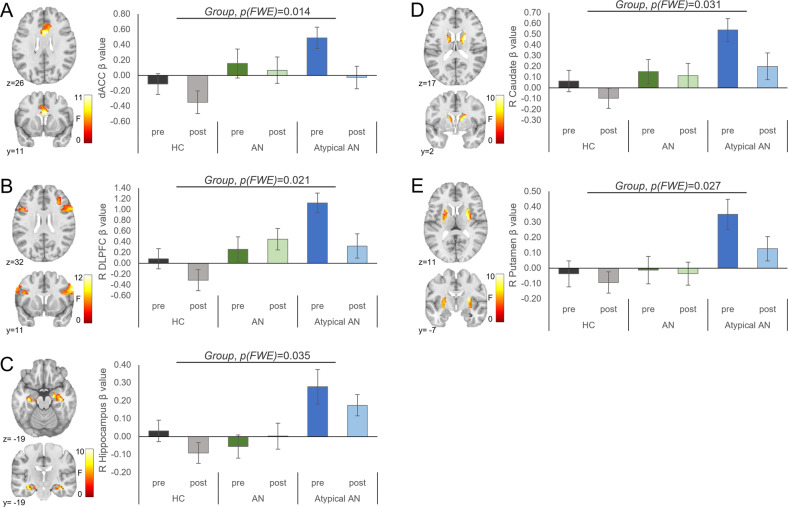
Results of Model 2 showing significant main effects of Group (healthy control [HC]; anorexia nervosa [AN]; atypical AN) in blood oxygenation level-dependent (BOLD) activation to high-calorie foods versus objects in a priori regions of interest (ROIs). BOLD activation differed between groups (HC: *n* = 34; AN: *n* = 34; Atypical AN: *n* = 25) in the (**A**) dorsal anterior cingulate cortex (dACC), (**B**) right dorsolateral prefrontal cortex (DLPFC), (**C**) right hippocampus, (**D**) right caudate, and (**E**) right putamen. The *F* scale and *P* values reflect the main effect of Group from the 3 (Group) × 2 (Appetitive State) analysis of covariance (with age and estradiol levels at the time of testing as covariates). Statistical thresholding reflects small-volume correction (SVC) within an anatomically-defined bilateral ROI at *P*(FWE-corrected) < 0.05. Statistical maps for BOLD activation are overlaid on a normalized canonical image (Montreal Neurological Institute [MNI] ICBM 152 nonlinear asymmetric T1 template) with SPM color map corresponding to relative *F* value. Coordinates (*y*, *z*) are presented in MNI space, with y corresponding to the coronal plane and *z* to the axial plane. Bar graph (right) depicts mean *β* values for each (sub-)group and Appetitive State ± SEM.

As hypothesized, we found main effects for Group for BOLD activation in the dACC, right DLPFC, right hippocampus, right caudate, and right putamen (no significant effects in the anterior insula, hypothalamus, amygdala, OFC, or NAcc) (Fig. 3); and main effects of Appetitive State for BOLD activation in the left NAcc only. We found no Group by Appetitive State interaction effects in any a priori ROIs (Table 2). Post hoc pairwise comparisons for the main effects of Group showed that dACC BOLD activation was higher in the AtypAN subgroup compared to the HC group (mean difference [based on estimated marginal means]±*SE*: 0.53 ± 0.19, *P* = 0.016) and higher in the AN subgroup compared to HC group (0.46 ± 0.18, *P* = 0.038), while AtypAN and AN subgroups did not differ (0.07 ± 0.18, *P* = 1.0). The right DLPFC was more activated in the AtypAN subgroup compared to the HC group (0.83 ± .25, *P* = 0.003), while no differences were observed between AtypAN and AN as well as AN and HC (sub-)groups (0.34 ± 0.24, *P* = 0.492 and 0.49 ± 0.24, *P* = 0.134, respectively). Right hippocampus BOLD activation was higher in the AtypAN subgroup compared to HC and AN (sub-)groups (0.25 ± 0.07, *P* = 0.003 and 0.25 ± 0.07, *P* = 0.002, respectively) with no differences between AN and HC (sub-)groups (−0.01 ± 0.07, *P* = 1.0). Right caudate activation was greater in the AtypAN subgroup compared to HC group (0.41 ± 0.14, *P* = 0.009), while no differences were observed between AtypAN and AN or the AN and HC (sub-)groups (0.21 ± 0.14, *P* = 0.366 and 0.20 ± 0.13, *P* = 0.396, respectively). Finally, right putamen BOLD activation was higher in the AtypAN subgroup compared to the HC group (0.32 ± 0.10, *P* = 0.007) with no significant differences between AtypAN and AN or AN and HC (sub-)groups (0.25 ± 0.10, *P* = 0.051 and 0.07 ± 0.10, *P* = 1.0, respectively).

## Discussion

Adolescent and young adult females with AN and AtypAN demonstrated hyperactivation of certain brain regions known to be involved in appetite and food intake when viewing images of high-calorie foods versus objects, relative to HC. While individuals with EDs and HC both showed robust response to images of high-calorie foods, those with EDs showed increased BOLD activations in neural regions that have been associated with cognitive control and reward processing relative to HC. We found both main Group *and* Appetitive State effects, but no interaction effects (counter to our hypothesis), suggesting that these group effects are robust, whereas the pre-to-postmeal attenuation was similar in both groups. The group effects that emerged were specific rather than occurring across all tested ROIs involved in the processing of high-calorie food cues. We saw no between-group differences in activation in the insula or hypothalamus— both regions involved in receiving and relaying sensory input, or in activation in the amygdala, OFC, or NAcc —regions responsible for registering primary emotions including reward. By contrast, we observed a consistent pattern of between-group differences in dACC, DLPFC, hippocampus, caudate, and putamen.

Greater neural activation in the dACC and DLPFC—brain regions typically associated with cognitive control processing in individuals with AN/AtypAN (vs. HC) in the context of food images is consistent with previous findings demonstrating that engagement of cognitive control is required for behavioral adaptation to changing environmental demands [21, 22]. Yet, it extends previous results, showing that BOLD activations in regions associated with cognitive control were increased even during a passive viewing condition, namely, when cognitive control was not explicitly required. Further, the dACC, while involved in cognitive control, has also been implicated in reward valuation and modulation of reward responsiveness as it has direct projections to the ventral tegmental area, which regulates reward consumption and behavior [38, 39]. This finding of increased dACC and DLPFC activation raises the possibility that cognitive control is maladaptively over-engaged in those with AN and AtypAN, through automatic activation triggered by ED-relevant food reward stimuli.

Interestingly, the observed greater BOLD activation in certain regions that have been associated with reward processing in female adolescents and young adults with AN/AtypAN (vs. HC) in response to high-calorie food (vs. non-food) images—namely the hippocampus, caudate, and putamen—contrasted with previous findings from our group showing less BOLD activation in reward-related regions in women with AN (vs. HC) employing the same fMRI paradigm [20]. Notably, this earlier study included a smaller sample size and was limited to amenorrheic *adult* females with restricting-type AN, which might account for the differences. Indeed, in the current study, differences within the ED clinical subgroups emerged as well, elucidating possible neurobiological correlates of divergent symptom presentation. While both AtypAN and AN subgroups showed increased activation in dACC relative to HC, AtypAN further had increased activation in the right DLPFC, right hippocampus, right caudate, and right putamen activation relative to HC, whereas in the subgroup analyses, AN and HC did not differ significantly on activation in these regions. Furthermore, AtypAN showed increased neural activation specifically in the hippocampus relative to both AN and HC (sub-)groups. The caudate and the putamen are involved in modulating behavioral reward response, and the hippocampus is implicated in food regulation and has been described both as housing food reward memories and inhibiting eating behavior [7]. Taken together, these findings suggest greater recruitment of both regions that are involved in cognitive control *and* reward processing in the AtypAN subgroup compared to HC and in hippocampus activation relative to the AN subgroup.

These findings of hyperactivation of brain regions involved in cognitive control and reward-related processing in response to passive viewing of high-calorie food images across the ED sample, and particularly in AtypAN, suggest that dysfunctional appetite and food intake pathways play a role in symptom presentation in EDs characterized by driven food restriction. Observed alterations in neural activation in brain regions that have been implicated in reward and cognitive control provide evidence to support existing models of aberrant brain activation patterns underlying ED psychopathology [40, 41]. Our findings of increased activation in brain regions involved in cognitive control in AN and AtypAN may reflect the recruitment of effortful cognitive control to resist eating. In AtypAN, the increased recruitment of brain regions involved in cognitive control in the context of greater relative activation of regions involved in reward processing in response to food stimuli raises the possibility that restriction may be particularly effortful for those who are not as low weight [42]. Whether hyperactivation of regions involved in cognitive control and reward-related processing leads to the relatively higher weight in AtypAN or longitudinal diagnostic crossover from restricting to binge-eating/purging type illnesses [43–45] warrants future study.

Importantly, while the food cue paradigm we used is well-established and robustly activates brain regions involved in appetite and food intake, the passive nature of the task makes conceptualization of the neural regions that were differentially activated across groups as reflecting cognitive control and reward inferential rather than conclusive [16]. It is conceivable that the observed neural activations could also reflect surprise (e.g., to changes in stimulus categories at the transition between stimulus blocks), as processing of violated expectations activates a network including ventral striatum, insula, and PFC [46, 47], and/or fear and avoidance, which might probe the fear neurocircuitry when viewing images of (high-calorie) foods [48]. Notably, activation of the DLPFC has been shown to be associated with inhibition of appetitive responses (e.g., [21, 49–54]). Likewise, altered PFC activation and increased hippocampal and striatal activation demonstrated in response to visual food cues in those with obesity compared to controls have been argued to reflect impaired cognitive control and increased reward responsiveness [55]. Indeed, we selected a well-established *passive* viewing of food paradigm rather than an *active* task to focus on automatic processing that may differentiate those with AN/AtypAN from HC. By definition, those with AN/AtypAN exhibit behaviors that drive weight loss or maintain low weight; the degree to which abnormal neural activation underlies restriction even when actual food is not present speaks to the automaticity of neural response and may offer novel insights for treatment development.

Study strengths include the interdisciplinary assessment of food cue processing via fMRI. Repetition of measures from pre- to post-standardized meal allowed for a nuanced examination of eating behavior in this population. Inclusion of a well-phenotyped, heterogeneous adolescent and young adult sample of females with AN and AtypAN allowed for investigation across and then within clinical presentation compared to HC. However, important limitations warrant acknowledgement. First, as noted, our selected fMRI paradigm was a passive viewing task. Future studies should include both active and passive food paradigms as well as behavioral assessments of non-food-related cognitive control and reward processing to comprehensively test questions related to over-engagement of cognitive control and reward-related regions in this population [16]. Likewise, these studies should also evaluate surprise or fear that may help to interrogate alternative hypotheses involving activation in these neural regions in response to food cues. Second, while the variance in ED symptomatology was a strength of our study and we were able to split the full ED group by degree of low weight, with low body weight (defined as ≤90% of expected body weight) being a study inclusion criterion, we could not examine the full weight spectrum of individuals captured by the AtypAN diagnosis. Further, our cohort of individuals in the BP-type subgroup was small (*n* = 16), rendering that set of findings exploratory (see Supplementary Materials). Future studies should include individuals with AtypAN across the weight spectrum, and a larger sample of individuals with a BP-type presentation. Finally, in order to keep the assessment measures consistent across the sample, we used the K-SADS-PL (originally designed for use with children and adolescents) with young adults.

Our results lay the groundwork for future investigations based on large samples to further unveil the shared or symptom-specific characteristics of food-cued neural activation in those with eating disorders, and which combines these neural data with other biologic modulators of appetite data (e.g., endocrine signaling) and their synergistic interplay or disruption thereof in explaining the clinical phenotypes of these complex conditions. This line of research could guide the development of targeted interventions to interrupt symptom consolidation.

## Supplementary information

Supplementary Materials

Supplementary Figure
