# Supplementary material for: Neural activation of regions involved in food reward and cognitive control in young females with anorexia nervosa and atypical anorexia nervosa versus healthy controls
Source: Transl Psychiatry. 2023 Jun 23;13:220. doi: 10.1038/s41398-023-02494-3 (PMC10290133; doi:10.1038/s41398-023-02494-3)
Supplement: Supplementary file 1 — Supplementary Materials [file 41398_2023_2494_MOESM1_ESM.docx]

**Neural activation of regions involved in food reward and cognitive control in young females with anorexia nervosa and atypical anorexia nervosa versus healthy controls – Supplementary materials**

**Materials and Methods: Additional Details**

*Functional MRI Data Acquisition*

Whole-brain functional imaging was performed using a gradient-echo EPI pulse sequence (32 contiguous oblique-axial slices, 4-mm thick, TR/TE=2530/30 ms, flip angle=85°, FOV=216x216 mm, 120 total images per run). A sagittal 3D T1-weighted MPRAGE sequence was acquired (TR/TE=2,530/3.43 ms, flip angle=7°, FOV=256x256 mm, effective slice thickness=1.33 mm with 128 slices) for co-registration between structural and functional datasets.

*Data Processing and Statistical Analysis*

The anatomical regions of interest (ROIs) were defined using the Automated Anatomical Labeling atlas version 3 (AAL3; 1). Within each ROI, we report clusters that (a) were initially significant at *p*<0.05 uncorrected, (b) met or exceed an extent threshold of *k*=5 for the nucleus accumbens and *k*=20 for all other ROIs, and (c) additionally met the peak-level threshold of *p*<0.05, FWE-corrected for the ROI. In addition to hypotheses for *a priori* ROIs, group differences in whole-brain activation (i.e., not restricted to *a priori* ROI masks) were examined at a conservative threshold to guard against spurious findings: significant at *p*<0.001, uncorrected and met a whole-brain cluster-level threshold of *p*<0.05, FWE-corrected. For clusters in *a priori* ROIs reaching statistical significance for the above models, parameter estimates were extracted with the REX toolbox (2) for visual display and plotting.

For data collected at each session (premeal, postmeal), preprocessing steps included realignment and geometric unwarping using magnitude and phase images from the fieldmap, slice-time correction, EPI coregistration to the subject’s segmented, skull-stripped, bias-corrected T1 image, normalization to Montreal Neurological Institute (MNI) space with resampling to 2 mm isotropic using 4^th^ degree B-spline interpolation, and smoothing with a 6-mm isotropic full width at half-maximum (FWHM) Gaussian kernel. Each run of EPI data was evaluated for motion using two approaches. First, the degree of linear movement was assessed and classified according to a threshold of 3 mm linear movement in the orthogonal planes. Second, the ART toolbox (Artifact Detection Tools; www.nitrc.org/projects/artifact_detect) was used to detect outlier volumes based on image intensity relative to the global mean image (threshold: 3.5 *SD*) and motion (threshold: 0.8 mm, measured as scan-to-scan movement). Individual runs were excluded if they met either of the following criteria: 1) >3mm linear movement and >10% outliers; 2) >20% outliers. Additionally, if a subject had ≥3 runs within a session (premeal, postmeal) that were excluded according to these criteria, the subject’s dataset for that session was excluded. Based on these criteria, from our originally acquired data set of *N*=101 subjects, 8 participants (4 healthy controls [HC], 4 individuals with anorexia nervosa [AN]/atypical AN [AtypAN]) were excluded from the analyses. Following these exclusions, *n*=93 participants with usable data available were included in the analyses.

Statistical analysis of fMRI data focused on the high-calorie food versus object contrast, and repeated-measures analyses of covariance were applied for hypothesis testing. For subject-level analyses, three regressors were specified, according to images presented in the block design: high-calorie foods, low-calorie foods, and objects. Regressors were placed at the onset of each condition within a block with duration of 15 s and were convolved with the canonical hemodynamic response function. Global mean signal and motion outliers, detected using ART, were entered as nuisance regressors. Following general linear model estimation, the primary contrast (high-calorie foods vs. objects) was computed at the single-subject level.

**Results: Secondary Exploratory and Whole-Brain Analyses**

*Participant characteristics*

Comparing individuals with AN/AtypAN of the restricting type, binge/purge (BP) type, and HC, by definition both restricting-type and BP-type subgroups were lower weight than HC and had greater eating disorder (ED) psychopathology than the HC group. Restricting-type and BP-type subgroups did not differ in weight or in ED psychopathology from each other (**Supplementary Table 1**). Of the 16 individuals with binge-eating/purging presentations, three were engaging in binge-eating and purging, five were binge-eating only, and eight were purging only.

*Secondary exploratory Model 3: Restricting-type, BP-type, and HC*

We found main effects of Group for blood oxygenation level-dependent (BOLD) activation in the left anterior insula, dorsal anterior cingulate cortex (dACC), and bilateral dorsolateral prefrontal cortex (DLPFC) (no significant effects in hypothalamus, amygdala, OFC, hippocampus, NAcc, caudate, or putamen, ROIs) (**Supplementary Figure 1 and Supplementary Table 2**). We found no main effects of Appetitive State and no interaction effects in any of the *a priori* ROIs. Post-hoc pairwise comparisons for main effects of Group revealed that left anterior insula BOLD activation was higher in the BP-type subgroup compared to the HC group (0.32±0.09, p=0.001), while no differences were observed between BP-type and restricting-type as well as restricting-type and HC (sub-)groups (0.17±0.08, p=0.103 and 0.14±0.07, p=0.110, respectively). The dACC showed higher BOLD activation in the BP-type subgroup compared to the HC group (0.69±0.19, p=0.001) with no significant differences in BOLD activation between BP-type and restricting-type as well as restricting-type and HC (sub-)
groups (0.41±0.18, p=0.074 and 0.28±0.15, p=0.173, respectively). The right DLPFC (peak voxel: 25 38 35) showed more BOLD activation in the BP-type compared to both HC and restricting-type (sub-)groups (0.80±0.23, p=0.002 and 0.77±0.21, p=0.001, respectively), while restricting-type subgroup and HC group did not differ (0.03±0.18, p=1.0). Similarly, left DLPFC activation (peak voxel: -24 35 26) was higher in the BP-type compared to both HC and restricting-type (sub-)groups (0.85±0.20, p<0.001 and 0.71±0.19, p=0.001, respectively) with no observed difference between restricting-type subgroup and HC group (0.14±0.16, p=1.0). For the second cluster in the right DLPFC that showed a main effect of Group (peak voxel: 61 11 32), BOLD activation was higher in both BP-type and restricting-type subgroups compared to HC (0.79±0.27, p=0.013 and 0.71±0.21, p=0.003, respectively), while BP-type and restricting-type subgroups did not differ (0.08±0.25, p=1.0). Taken together, when the ED group was split by primary behavioral phenotype, we found bilateral premeal hyperactivation in the left anterior insula, dACC, and right and left DLPFC in those with the BP-type presentation compared to the restricting-type subgroup and HC group, suggesting the greatest activation of areas involved in cognitive cotnrol in the BP-type subgroup, while we found no differences here in BOLD activation of reward-related brain regions.

*Whole-brain analyses*

An examination of group differences in whole-brain activation using the contrast high-calorie foods versus objects (as employed for the ROI approach) yielded significant differences for all three models matching key results based on analysis of activation of *a priori* ROIs (**Supplementary Table 3**). Additional analyses of group differences in whole-brain activation based on the contrast low-calorie foods versus objects yielded significant main effects of Group in the occipital gyrus for all three models, and of Appetitive State in the hypothalamus in the comparison of ED versus HC groups (**Supplementary Table 4**).

**References**

1. Tzourio-Mazoyer N, Landeau B, Papathanassiou D, Crivello F, Etard O, Delcroix N *et al*. Automated anatomical labeling of activations in SPM using a macroscopic anatomical parcellation of the MNI MRI single-subject brain. *Neuroimage* 2002;15(1):273-289.

2. Whitfield-Gabrieli S. *Region of Interest Extraction (REX) Toolbox* (Boston, MA, 2009).

**Figure Legends**

**Supplementary Figure 1.** Results of the secondary exploratory Model 3 showing significant main effects of Group (healthy control [HC]; restricting-type; binge-eating/purging [BP]-type]) in blood oxygenation level-dependent (BOLD) activation to high-calorie foods versus objects in *a priori* regions of interest (ROIs). BOLD activation differed between groups (HC: *n*=34; restricting-type: *n*=34; BP-type: *n*=43) in the (A) left anterior insula, (B) dorsal anterior cingulate cortex (dACC), and separate clusters in the (C, E) right and (D) left dorsolateral prefrontal cortex (DLPFC). The *F* scale and *p* values reflect the main effect of Group from the 3 (Group) x 2 (Appetitive State) analysis of covariance (with age and estradiol levels at the time of testing as covariates). Statistical thresholding reflects small volume correction (SVC) within an anatomically-defined bilateral ROI at *p*(FWE-corrected)<0.05. Statistical maps for BOLD activation are overlaid on a normalized canonical image (Montreal Neurological Institute [MNI] ICBM 152 nonlinear asymmetric T1 template) with SPM color map corresponding to relative F value. Coordinates (y,z) are presented in MNI space, with y corresponding to the coronal plane and z to the axial plane. Bar graph (right) depicts mean β values for each (sub-)group and appetitive state±*SEM*.

**Supplementary Table 1.** Participant characteristics for healthy control (HC), restricting-type, and binge/purge-(BP-)type (sub-)groups

| **Characteristic** | **HC group (*n*=34)** | | **Restricting-type subgroup (*n*=43)** | | **BP-type subgroup (*n*=16)** | | **HC, restricting-type, and BP-type (sub-)groups** | |
| --- | --- | --- | --- | --- | --- | --- | --- | --- |
|  | *n* | Mean±*SD*/ n (%) | *n* | Mean±*SD*/ n (%) | *n* | Mean±*SD*/ n (%) | *p* | η_p_^2^ |
| Age (years) | 34 | 18.2±2.9 | 43 | 18.8±2.9 | 16 | 19.7±1.8 | 0.169 | 0.04 |
| Race | 34 |  | 43 |  | 16 |  |  |  |
| American Indian/ Alaska Native |  | 0 (0.0) |  | 0 (0.0) |  | 0 (0.0) |  |  |
| Black/ African American |  | 0 (0.0) |  | 0 (0.0) |  | 0 (0.0) |  |  |
| Asian |  | 5 (14.7) |  | 4 (9.3) |  | 6 (37.5) |  |  |
| White |  | 28 (82.4) |  | 38 (88.4) |  | 10 (62.5) |  |  |
| Other |  | 1 (2.9) |  | 1 (2.3) |  | 0 (0.0) | 0.104 |  |
| Ethnicity | 34 |  | 43 |  | 16 |  |  |  |
| Hispanic/Latino |  | 1 (2.9) |  | 2 (4.7) |  | 2 (12.5) |  |  |
| Non-Hispanic/Latino |  | 33 (97.1) |  | 41 (95.3) |  | 14 (87.5) | 0.318 |  |
| Time since self-diagnosis (years) | n/a | n/a | 43 | 4.3±3.4 | 15 | 4.7±4.2 | –– | –– |
| BMI (kg/m²) | 34 | 21.6±1.7 | 43 | 17.5±1.5 | 16 | 18.0±1.2 | **<0.001**^1,2^ | 0.62 |
| BMI z-score (if <20 years of age) | 24 | 0.2±0.5 | 24 | -1.5±0.9 | 8 | -1.5±0.7 | **<0.001**^1,2^ | 0.60 |
| EDE |  |  |  |  |  |  |  |  |
| Restraint | 34 | 0.0±0.0 | 43 | 2.8±1.6 | 16 | 3.1±1.2 | **<0.001**^1,2^ | 0.57 |
| Eating Concern | 34 | 0.0±0.0 | 43 | 1.9±1.4 | 15 | 1.9±1.4 | **<0.001**^1,2^ | 0.42 |
| Shape Concern | 34 | 0.1±0.2 | 43 | 3.2±2.0 | 16 | 3.6±2.0 | **<0.001**^1,2^ | 0.50 |
| Weight Concern | 34 | 0.04±0.1 | 43 | 3.1±1.9 | 16 | 3.0±1.8 | **<0.001**^1,2^ | 0.50 |
| Global Score | 34 | 0.03±0.1 | 43 | 2.7±1.6 | 15 | 2.8±1.3 | **<0.001**^1,2^ | 0.56 |
| Estradiol (pg/mL) | 34 | 61.3±50.9 | 43 | 40.5±49.3 | 16 | 42.9±39.6 |  |  |
| Log_10_-estradiol | 34 | 1.7±0.3 | 43 | 1.3±0.6 | 16 | 1.4±0.5 | **0.009**^1^ | 0.10 |

*Note.* Significant *p*-values are bolded. ^1^HC group≠Restricting-type subgroup, *p*<0.05. ^2^HC group≠BP-type subgroup, *p*<0.05. BMI, body mass index; EDE, Eating Disorder Examination.

**Supplementary Table 2.** Significant main effects and interactions of Group and Appetitive State (premeal, postmeal) for the secondary exploratory model (healthy control [HC] vs. restricting-type vs. binge-purge-[BP]-type [sub-]groups) for blood oxygenation level-dependent (BOLD) activation to high-calorie foods versus objects in *a priori* primary and secondary regions of interest with age and estradiol levels at the time of testing as covariates

|  | **R/L**^a^ | **Peak *F* value** | **k(E)**^b^ | ***p* (FWE_corr_)**^c^ | **x**^d^ | **y** | **z** | **Also meets whole-brain thresholds** |
| --- | --- | --- | --- | --- | --- | --- | --- | --- |
| **Model 3: HC (*n*=34), restricting-type (*n*=43), and BP-type (*n*=16) (sub-)groups** | | | | | | | | |
| **Main effect of Group** |  |  |  |  |  |  |  |  |
| Anterior insula | L | 9.88 | 59 | 0.028 | -27 | 17 | 14 |  |
| dACC | -- | 10.76 | 500 | 0.011 | 0 | 5 | 29 |  |
| DLPFC | R | 18.71 | 298 | <0.001 | 24 | 38 | 35 | x |
|  | L | 14.92 | 270 | 0.001 | -24 | 35 | 26 | x |
|  | R | 10.85 | 76 | 0.033 | 63 | 11 | 32 |  |
| **Main effect of Appetitive State** |  |  |  |  |  |  |  |  |
| *No significant clusters* |  |  |  |  |  |  |  |  |
| **Group × Appetitive State interaction** |  |  |  |  |  |  |  |  |
| *No significant clusters* |  |  |  |  |  |  |  |  |

*Note.* ^a^R/L denotes hemisphere in which peak voxel within each cluster was localized. ^b^Cluster size (contiguous voxels). ^c^Statistical significance was assessed at *p*<0.05 FWE-corrected using small-volume correction with a minimum cluster size of *k*=5 in the nucleus accumbens and *k*=20 in all other regions of interest. ^d^Coordinates are presented in Montreal Neurological Institute (MNI) space. dACC, dorsal anterior cingulate cortex; DLPFC, dorsolateral prefrontal cortex.

**Supplementary Table 3.** Significant main effects and interactions of Group and Appetitive State (premeal, postmeal) for the two primary models (1: Healthy control [HC] vs. eating disorder [ED] groups; 2: HC vs. anorexia nervosa [AN] vs. atypical AN [AtypAN] [sub-]groups) and the secondary exploratory model (HC vs. restricting-type vs. binge-purge [BP]-type [sub-]groups) for blood oxygenation level-dependent (BOLD) activation to high-calorie foods versus objects in whole-brain regions with age and estradiol levels at the time of testing as covariates

|  | **R/L**^a^ | **Peak *F* value** | **k(E)**^b^ | ***p* (cluster-level FWE_corr_)**^c^ | **x**^d^ | **y** | **z** |
| --- | --- | --- | --- | --- | --- | --- | --- |
| **Model 1: HC (*n*=34) versus ED (*n*=59) groups** |  |  |  |  |  |  |  |
| **Main effect of Group** |  |  |  |  |  |  |  |
| Inferior temporal gyrus^e^ | R | 29.5 | 161 | <0.001 | 42 | -76 | -4 |
| Supramarginal gyrus | L | 29.22 | 155 | 0.001 | -45 | -31 | 29 |
| Superior frontal gyrus, lateral part | R | 20.97 | 102 | 0.007 | 12 | 14 | 68 |
| dACC/cingulate gyrus, dorsal part | -- | 19.99 | 100 | 0.007 | 0 | 11 | 26 |
| Middle temporal gyrus | L | 19.18 | 99 | 0.008 | -39 | -58 | -10 |
| Occipital gyrus | L | 17.95 | 121 | 0.003 | -30 | -85 | -16 |
| Superior parietal lobule | L | 17.39 | 91 | 0.012 | -33 | -61 | 56 |
| **Main effect of Appetitive State** |  |  |  |  |  |  |  |
| *No significant clusters* |  |  |  |  |  |  |  |
| **Group × Appetitive State interaction** |  |  |  |  |  |  |  |
| *No significant clusters* |  |  |  |  |  |  |  |
| **Model 2: HC (*n*=34), AN (*n*=34), and AtypAN (*n*=25) (sub-)groups** |  |  |  |  |  |  |  |
| **Main effect of Group** |  |  |  |  |  |  |  |
| Inferior temporal gyrus | R | 20.02 | 552 | <0.001 | 42 | -76 | -7 |
| Parietal operculum | L | 16.08 | 101 | 0.004 | -48 | -28 | 29 |
| Inferior temporal gyrus | L | 15.47 | 462 | <0.001 | -39 | -52 | -10 |
| dACC/cingulate gyrus, dorsal part | -- | 10.44 | 60 | 0.039 | 0 | 11 | 26 |
| Pallidum | R | 11.11 | 120 | 0.001 | 15 | 2 | 5 |
| **Main effect of Appetitive State** |  |  |  |  |  |  |  |
| *No significant clusters* |  |  |  |  |  |  |  |
| **Group × Appetitive State interaction** |  |  |  |  |  |  |  |
| *No significant clusters* |  |  |  |  |  |  |  |
| **Model 3: HC (*n*=34), restricting-type (*n*=43), and BP-type (*n*=16) (sub-)groups** | | | | | | | |
| **Main effect of Group** |  |  |  |  |  |  |  |
| White matter | L | 21.39 | 328 | <0.001 | -21 | -49 | 32 |
| DLPFC/Superior frontal gyrus, lateral part | R | 18.71 | 705 | <0.001 | 24 | 38 | 35 |
| DLPFC/Superior frontal gyrus, lateral part | L | 14.92 | 248 | <0.001 | -24 | 35 | 26 |
| Inferior temporal gyrus | R | 14.7 | 108 | 0.002 | 42 | -76 | -4 |
| White matter | R | 12.17 | 294 | <0.001 | 30 | -25 | 35 |
| Superior frontal gyrus, lateral part | R | 11 | 60 | 0.034 | 6 | 11 | 65 |
| **Main effect of Appetitive State** |  |  |  |  |  |  |  |
| *No significant clusters* |  |  |  |  |  |  |  |
| **Group × Appetitive State interaction** |  |  |  |  |  |  |  |
| *No significant clusters* |  |  |  |  |  |  |  |

*Note.* ^a^R/L denotes hemisphere in which peak voxel within each cluster was localized. ^b^Cluster size (contiguous voxels). ^c^Statistical significance was assessed at cluster-level *p*<0.05 FWE-corrected across whole brain with a minimum cluster size of *k*=20. ^d^Coordinates are presented in Montreal Neurological Institute (MNI) space. ^e^Regions mapped using the Mai et al. *Atlas of the human brain*, 3^rd^ Ed. (2008). dACC, dorsal anterior cingulate cortex; DLPFC, dorsolateral prefrontal cortex.

**Supplementary Table 4.** Significant main effects and interactions of Group and Appetitive State (premeal, postmeal) for the two primary models (1: Healthy control [HC] vs. eating disorder [ED] groups; 2: HC vs. anorexia nervosa [AN] vs. atypical AN [AtypAN] [sub-]groups) and the secondary exploratory model (HC vs. restricting-type vs. binge-purge [BP]-type [sub-]groups) for blood oxygenation level-dependent (BOLD) activation to low-calorie foods versus objects in whole-brain regions with age and estradiol levels at the time of testing as covariates

|  | **R/L**^a^ | **Peak *F* value** | **k(E)**^b^ | ***p* (cluster-level FWE_corr_)**^c^ | **x**^d^ | **y** | **z** |
| --- | --- | --- | --- | --- | --- | --- | --- |
| **Model 1: HC (*n*=34) versus ED (*n*=59) groups** |  |  |  |  |  |  |  |
| **Main effect of Group** |  |  |  |  |  |  |  |
| Occipital gyrus | L | 25.01 | 157 | <0.001 | -39 | -94 | -1 |
|  | R | 20.95 | 70 | 0.023 | 42 | -76 | -4 |
| **Main effect of Appetitive State** |  |  |  |  |  |  |  |
| Hypothalamus | R | 24.36 | 73 | 0.019 | 9 | 2 | -13 |
| **Group × Appetitive State interaction** |  |  |  |  |  |  |  |
| *No significant clusters* |  |  |  |  |  |  |  |
| **Model 2: HC (*n*=34), AN (*n*=34), and AtypAN (*n*=25) (sub-)groups** |  |  |  |  |  |  |  |
| **Main effect of Group** |  |  |  |  |  |  |  |
| Occipital gyrus | R | 18.08 | 341 | <0.001 | 45 | -82 | -16 |
|  | L | 17.37 | 384 | <0.001 | -36 | -94 | -7 |
| **Main effect of Appetitive State** |  |  |  |  |  |  |  |
| *No significant clusters* |  |  |  |  |  |  |  |
| **Group × Appetitive State interaction** |  |  |  |  |  |  |  |
| *No significant clusters* |  |  |  |  |  |  |  |
| **Model 3: HC (*n*=34), restricting-type (*n*=43), and BP-type (*n*=16) (sub-)groups** | | | | | | | |
| **Main effect of Group** |  |  |  |  |  |  |  |
| Occipital gyrus | L | 12.67 | 59 | 0.033 | -39 | -94 | -1 |
| **Main effect of Appetitive State** |  |  |  |  |  |  |  |
| *No significant clusters* |  |  |  |  |  |  |  |
| **Group × Appetitive State interaction** |  |  |  |  |  |  |  |
| *No significant clusters* |  |  |  |  |  |  |  |

*Note.* ^a^R/L denotes hemisphere in which peak voxel within each cluster was localized. ^b^Cluster size (contiguous voxels). ^c^Statistical significance was assessed at cluster-level *p*<0.05 FWE-corrected across whole brain with a minimum cluster size of *k*=20. ^d^Coordinates are presented in Montreal Neurological Institute (MNI) space. ^e^Regions mapped using the Mai et al. *Atlas of the human brain*, 3^rd^ Ed. (2008). dACC, dorsal anterior cingulate cortex; DLPFC, dorsolateral prefrontal cortex.
