# Supplementary figures and images for: Neural activation of regions involved in food reward and cognitive control in young females with anorexia nervosa and atypical anorexia nervosa versus healthy controls
Source: Transl Psychiatry. 2023 Jun 23;13:220. doi: 10.1038/s41398-023-02494-3 (PMC10290133; doi:10.1038/s41398-023-02494-3)

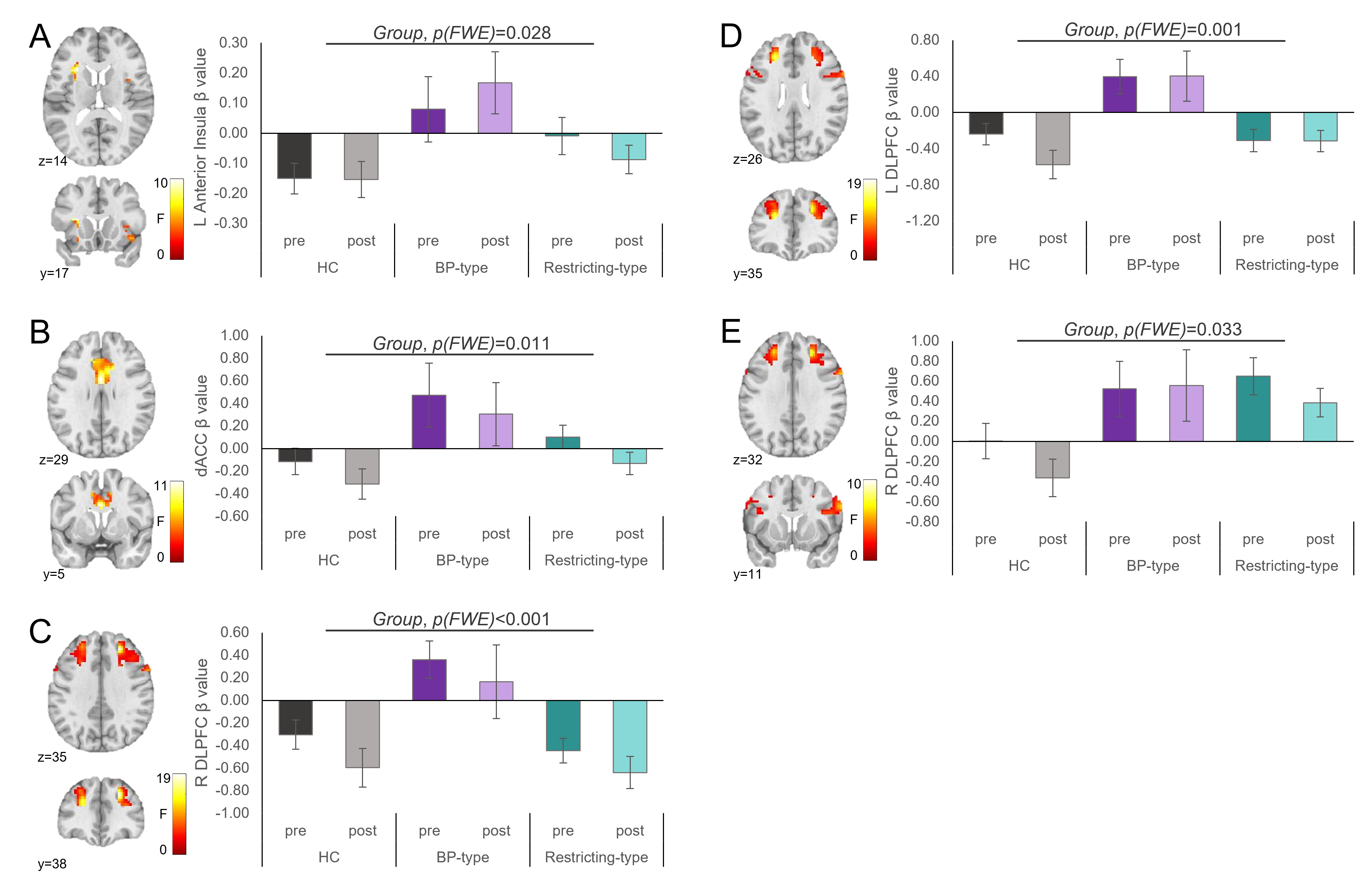

Supplement: Supplementary file 2 — Supplementary Figure [file 41398_2023_2494_MOESM2_ESM.png]
